# Post-conization cervical stenosis treated with silicone catheter in microinvasive cervical cancer patient: A case report

**DOI:** 10.1016/j.ijscr.2020.02.005

**Published:** 2020-02-06

**Authors:** Angela Musella, Giusi Santangelo, Laura Vertechy, Anna Di Pinto, Carolina Maria Sassu, Margherita Fischetti, Violante Di Donato, Giorgia Perniola, Innocenza Palaia, Pierluigi Benedetti Panici

**Affiliations:** Department of Maternal, Infantile, and Urological Sciences, University of Rome “Sapienza”, Umberto I Hospital, Viale del Policlinico 155, 00161 Rome, Italy

**Keywords:** Vaginal trachelectomy, Early cervical cancer, Cervical stenosis, Silicon urethral catheter

## Abstract

**Introduction:**

Cervical stenosis can represent a hard complication to treat after conization for microinvasive cervical cancer.

**Presentation of case:**

A young woman with cervical stenosis post-trachelectomy for a microinvasive cervical cancer came to our Department. We introduced a silicone catheter of 18 French in cervical canal. The catheter was removed after 20 days.

The procedure was resolutive.

**Discussion:**

Cervical stenosis is one of the most frequent complication of conization. Among the different described techniques proposed in literature, we would here report the successful use of a silicon urethral catheter into the cervical canal.

**Conclusion:**

This method can be an easy and cheap procedure to take in consideration.

## Highlights

• Cervical Cancer patient experienced a Cervical stenosis after conization.
• Cervical stenosis is one of the most frequent complication of conization.
• The use of a silicon urethral catheter into the cervical canal is a resolutive option.

## 1 Background

Microinvasive cervical cancer without LVSI can be safely managed with conization or simple trachelectomy to preserve fertility [1].

The most frequent long-term complications after conization are dysmenorrhea, dyspareunia, irregular menstruation or intermenstrual bleeding and cervical-isthmic stenosis. The incidence of cervical stenosis after conization is reported to be 1.6–17 % [[2], [3], [4], [5], [6], [7]]. There is no standard treatment for cervical stenosis in the literature.

The work has been reported in line with the SCARE criteria [8].

## 2 Case presentation

We report, with the patient's informed consent, one case of a young nulliparous patients with microinvasive cervical cancer, who experienced isthmic stenosis after conization, treated with the use of a silicone catheter of 18 French.

In March 2017, a 32-year-old Caucasian woman was referred to our Department for abnormal uterine bleeding. Gynecological examination with colposcopy showed a suspicious aceto-white zone that stain with Lugol solution on the anterior wall of cervix uteri. A biopsy of the lesion was performed which a squamous cell carcinoma histology. Subsequent instrumental investigations (MRI/PET-CT) were negative for extensive disease or lymphadenopathies. Considering the patient’s strong desire of future fertility, after a counselling on the risks and benefits of radical versus conservative surgery, she decided for a conservative treatment with electrosurgical conization. Histopathologic report confirmed a microinvasive squamous cell carcinoma, 5 mm in extension and 2 mm in stromal invasion, FIGO stage Ia1, without lymph vascular space involvement. Four weeks after the procedure, the patient complained amenorrhea and pelvic pain. A pelvic transvaginal 3D ultrasound suggested hematometra.

Hysteroscopy was than performed revealing a normal uterine cavity with abundant blood clots and a tight stenosis of the cervical canal. After the procedure we introduced a silicone catheter of 18 French in cervical canal. The catheter was removed after 20 days. Thirty days after the procedure the patient referred regular menstruation and absence of pelvic pain. After one year of follow up no disease recurrence was observed, and regular periods without pain or other symptoms have been reported at each scheduled visit.

## 3 Discussion

Cervical stenosis is one of the most frequent complication of conization. Cervical stenosis has been variously defined in literature. Some authors explain it as impossible to insert a cotton swab [9] or a Hegar dilator into the cervical canal [10], others consider cervical stenosis only when associated with hematometra or secondary amenorrhea [11]. Symptoms may occur in 50 % of cases, particularly in premenopausal women, including amenorrhea, dysmenorrhea, pathological bleeding and infertility. However, some of women develop stenosis of the cervical canal several months postoperatively, after having resumed normal menstruation.

Therefore, the reported incidence of cervical stenosis after conization varies from 4 % to 17 % [[12], [13], [14], [15], [16]]. Several strategies have been used in the management of this complication. Holmskov et al. reported the usefulness of Hegar cervical dilators in thirty-five patients out of 213 treated with conization [17]. Luesley et al. reported the favorable results of carbon dioxide laser vaporization to remove scar tissue after conization in patients with symptomatic cervical stenosis with success in sixteen (89 %) out of 18 patients [18]. Several studies have described successful treatment of severe cervical stenosis using different types of cervical stents. For example, urinary catheter [7] may be used, and Grund et al. reported a case of cervical stenosis successfully treated with a metal stent after conization to avoid recurrent stenosis and hematometra [18]. Whereas this self-expanding stent may be a useful device for severe stenosis, it is very expensive for routine use. Puzey et al. reported success with a copper IUD [12], and Nasu et al. a intrauterine contraceptive device and nylon threads that protruding through the stenotic cervical canal to provide the constitutive dilatation force to the stenotic tissue and allow the drainage of menstrual blood in four patients with a success rate of 100 % [10]. In our previous experience we described the Petit-Le Four cervical pessary as a good therapeutic option for the treatment of cervical stenosis [19].

## 4 Conclusion

In this paper we describe a simple, feasible not expensive method to treat cervical stenosis with a urethral silicon catheter soon after hysteroscopy. In our opinion this method could represents a valid alternative to avoid a recurrent cervical stenosis after conization, but more studies and patients are needed to confirm this hypothesis.

## Sources of funding

There aren’t funding sources.

## Ethical approval

The authors declare that all data were collected meeting ethical guidelines.

## Consent

Consent Written informed consent was obtained from the patient for publication of this case report.

## Author contribution

Conception, design, and coordination of the project: A. Musella.

Collection and assembly of data: G. Santangelo, L. Vertechy.

Manuscript drafting: A. Di Pinto and CM. Sassu, Margherita Fischetti.

Manuscript editing: Violante Di Donato and Giorgia Perniola.

Critical revision: A. Musella and Innocenza Palaia.

Final approval of the manuscript: P. Benedetti Panici.

## Registration of research studies

The authors declare that all data were collected meeting ethical guidelines.

Consent Written informed consent was obtained from the patient for publication of this case report.

## Guarantor

Angela Musella, Giusi Santangelo, Laura Vertechy, Anna Di Pinto, Carolina Maria Sassu, Margherita Fischetti, Violante Di Donato, Giorgia Perniola, Innocenza Palaia, Pierluigi Benedetti Panici.

## Provenance and peer review

Not commissioned, externally peer-reviewed.

## Declaration of Competing Interest

There are no conflicts of interest to declare for any of the authors.
